# Clearance of inflammatory cytokines in patients with septic acute kidney injury during renal replacement therapy using the EMiC2 filter (Clic-AKI study)

**DOI:** 10.1186/s13054-021-03476-x

**Published:** 2021-01-28

**Authors:** Nuttha Lumlertgul, Anna Hall, Luigi Camporota, Siobhan Crichton, Marlies Ostermann

**Affiliations:** 1 Department of Critical Care, Guy’s and St Thomas’ Hospital, King’s College London, NHS Foundation Trust, 249 Westminster Bridge Road, London, SE1 7EH UK; 2 Division of Nephrology and Excellence Centre for Critical Care Nephrology, King Chulalongkorn Memorial Hospital, Bangkok, Thailand; 3 Critical Care Nephrology Research Unit, Chulalongkorn University, Bangkok, Thailand; 4 Zorgsaam Terneuzen, Rotterdam, The Netherlands; 5 Medical Research Council Clinical Trials Unit, University College London, London, UK

**Keywords:** EMiC2 filter, Middle cut-off, High cut-off, Extracorporeal blood purification, Sepsis, Removal, Acute kidney injury, CRRT, Kidney replacement therapy

## Abstract

**Background:**

The EMiC2 membrane is a medium cut-off haemofilter (45 kiloDalton). Little is known regarding its efficacy in eliminating medium-sized cytokines in sepsis. This study aimed to explore the effects of continuous veno-venous haemodialysis (CVVHD) using the EMiC2 filter on cytokine clearance.

**Methods:**

This was a prospective observational study conducted in critically ill patients with sepsis and acute kidney injury requiring kidney replacement therapy. We measured concentrations of 12 cytokines [Interleukin (IL) IL-1β, IL-1α, IL-2, IL-4, IL-6, IL-8, IL-10, interferon (IFN)-γ, tumour necrosis factor (TNF)-α, vascular endothelial growth factor, monocyte chemoattractant protein (MCP)-1, epidermal growth factor (EGF)] in plasma at baseline (T0) and pre- and post-dialyzer at 1, 6, 24, and 48 h after CVVHD initiation and in the effluent fluid at corresponding time points. Outcomes were the effluent and adsorptive clearance rates, mass balances, and changes in serial serum concentrations.

**Results:**

Twelve patients were included in the final analysis. All cytokines except EGF concentrations declined over 48 h (*p* < 0.001). The effluent clearance rates were variable and ranged from negligible values for IL-2, IFN-γ, IL-1α, IL-1β, and EGF, to 19.0 ml/min for TNF-α. Negative or minimal adsorption was observed. The effluent and adsorptive clearance rates remained steady over time. The percentage of cytokine removal was low for most cytokines throughout the 48-h period.

**Conclusion:**

EMiC2-CVVHD achieved modest removal of most cytokines and demonstrated small to no adsorptive capacity despite a decline in plasma cytokine concentrations. This suggests that changes in plasma cytokine concentrations may not be solely influenced by extracorporeal removal.

*Trial registration*: NCT03231748, registered on 27th July 2017.

## Introduction

Sepsis is a life-threatening condition in which a dysregulated release of pro- and anti-inflammatory cytokines can lead to multiple organ failure and increased mortality [1]. Management of sepsis is supportive [2].

Critically ill patients with sepsis-associated acute kidney injury (AKI) requiring kidney replacement therapy (KRT) are at particularly increased risk of death [3]. However, KRT may offer the opportunity to provide extracorporeal blood purification therapy (BPT). With regards to the potential benefit of BPT, there are several hypotheses. The ‘peak concentration hypothesis’ proposes that during BPT excessive pro- or anti-inflammatory mediators are removed and plasma concentrations decrease to levels below a toxic threshold [4, 4]. The ‘cytokinetic theory’ proposes that cytokine removal creates a decreased cytokine gradient between tissues and the bloodstream and promotes leukocyte chemotaxis towards the infected tissues [6]. The ‘cytotoxic threshold immune modulation theory’ relates to the removal of cytokines from the cytokine-concentrated interstitium and tissues into the circulation [7]. Finally, a ‘cellular theory’ refers to removing leucocytes directly or through immune cell reprogramming [8, 9]. The proposed techniques include high-volume haemofiltration (HVHF), use of high cut-off (HCO) membrane and adsorption techniques, or plasmapheresis [10]. HCO haemofiltration with a cut-off up to 60 kDa has been shown to achieve higher cytokine clearance than conventional membranes (15–30 kDa), but the effects on clinical outcomes such as haemodynamic improvement, severity scores, and survival are inconclusive [11–17]. Besides, the concomitant loss of albumin, proteins, micronutrients, and antibiotics is a concern [18, 19].

The EMiC2 filter (Fresenius, Bad Homburg, Germany) is a polysulfone (PS)-based membrane with a cut-off of 45 kDa. Case reports showed reduction in serum k-free light chains and myoglobin levels with the EMiC2 filter, but actual removal by the filter was not investigated [20–23]. Other studies reported higher removal of kappa light chains (23 kDa), β2-microglobulin (17 kDa), myoglobin (17 kDa), IL-6, and IL-8 in patients receiving treatment with EMiC2 filters compared with standard high-flux membranes [24–27]. EMiC2-continuous veno-venous haemodialysis (CVVHD) was well-tolerated, and albumin loss was limited [28]. However, in-vitro data showed no adsorptive capacity of the EMiC2 filter compared with other membranes [29].

The exact role of the EMiC2 filter in the treatment of sepsis-associated AKI is unknown. Furthermore, the causal link between reduction in plasma cytokine concentration and extracorporeal cytokine removal has not been demonstrated [26, 28]. Before proceeding to a trial comparing the EMiC2 filter with other commercially available filters to manage sepsis-associated AKI, it is important to determine the characteristics and the actual magnitude of cytokine removal in vivo. In this pilot study, we aimed to measure the clearance of middle molecular weight cytokines using the EMiC2 filter in patients receiving CVVHD.

## Material and methods

This study was a prospective observational study in a 62-bed mixed tertiary-care intensive care unit (ICU) between June and September 2017. The study was approved by the Research Ethics Committee (16/LO/0313), registered on clinicaltrials.gov (NCT03231748) and conducted in accordance with the Declaration of Helsinki 2013. Written informed consent was obtained from all patients or their legal representatives.

### Subjects

Eligible patients were critically ill adult patients with acute kidney injury and sepsis in whom a decision had been made by the clinical team to start citrate-based CVVHD. Exclusion criteria were lack of consent, pre-existing dialysis-dependent kidney failure, life expectancy < 24 h, haemoglobin < 7 g/dL, and need for extracorporeal membrane oxygenation (ECMO).

### Kidney replacement therapy (KRT) setting

CVVHD was performed with the dialysis machine multifiltrate using the medium cut-off dialyzer EMiC2 and a bicarbonate-buffered dialysate (Fresenius Medical Care, Bad Homburg, Germany) at 25–30 ml/kg/h [30]. Regional citrate anticoagulation was used in all patients.

### Cytokine measurement

The concentrations of interleukin (IL)-2, IL-4, IL-6, IL-8, IL-10, vascular endothelial growth factor (VEGF), interferon gamma (IFN-γ), tumour necrosis factor alpha (TNF-α), IL-1alpha (IL-1α), IL-1beta (IL-1β), monocyte chemoattractant protein-1 (MCP-1), epidermal growth factor (EGF) were measured before initiation of CVVHD (T0) and pre- and post-dialyzer during CVVHD at 1, 6, 24, and 48 h (T1, T6, T24, and T48, respectively) (Additional file 1). These molecules were also measured in the effluent at the same time points (T1, T6, T24, and T48). If CVVHD had to be temporarily discontinued, sampling was performed 1–2 h after CVVHD was re-started using the same circuit. In case, a circuit change was necessary within the first 24 h of the study, sampling was re-commenced de novo with the new filter. If a further filter change was necessary within the first 24 h, the patient was withdrawn from the study.

### Laboratory analyses

Blood and effluent samples were centrifuged at 3000 rpm for 15 min and stored in a − 80 °C freezer until batch analysis at the end of the study. The cytokine concentrations were determined by electro-chemiluminescent immunoassay (ECLIA) method using an Evidence Investigator Bioship system (Randox Laboratories Limited, the United Kingdom).

### Outcomes

The primary outcome of interest was cytokine clearance during EMiC2-based CVVHD. The secondary endpoints were adsorption by the EMiC2 filter, changes in cytokine concentrations in plasma, and reduction ratios of all cytokines over 48 h.

#### Clearances

*Effluent clearance (Cl*_eff_*)* at each sampling time point was estimated using the following equation [31]:

1 $${\text{Cl}}_{{{\text{eff}} }} \left( {\frac{{{\text{ml}}}}{\min }} \right) = \frac{{Q_{{{\text{df}}}} }}{60} \times \frac{{{\text{Ceffluent}}}}{{\left( {{\text{Cpredialyzer}} + {\text{Cpostdialyzer}}} \right)/2}}$$

where *Q*_df_ represents dialysate flow rate (ml/h).

*Adsorptive clearance (K*_ad_*)* was calculated as:

2 $${\text{Kad}} \left( {\frac{{{\text{ml}}}}{\min }} \right) = \frac{{M_{{{\text{ad}}}} }}{{{\text{Cpredialyzer}}}}$$

*M*_ad_ represents mass removal rate by membrane adsorption (pg/min) (see below):

The average hourly effluent clearance (Cl_total_) during the study period was calculated using the following formula:

3 $$\begin{aligned}&{\text{Cl}}_{{{\text{total}}}} \left( {\frac{{{\text{ml}}}}{47}} \right) = \frac{{{\text{Cl}}1\;{\text{h}} + {\text{Cl}}6\;{\text{h}}}}{2} \times 5 \times 60 \\&\quad+ \frac{{{\text{Cl}}6\;{\text{h}} + {\text{Cl}}24\;{\text{h}}}}{2} \times 18 \times 60 + \frac{{{\text{Cl}}24\,{\text{h}} + {\text{Cl}}48\,{\text{h}}}}{2} \times 24 \times 60\end{aligned}$$

The period between starting CVVHD and the 1-h time point was not included due to the necessary equilibration process. Average clearance per minute (Cl_mean_) was determined from Cl_total_ as follows:

4 $${\text{Cl}}_{{{\text{mean}}}} \left( {\frac{{{\text{ml}}}}{\min }} \right) = \frac{{{\text{Cl}}_{{{\text{total}}}} }}{47 \times 60}$$

#### Mass balance

Mass balance equations describe the transport of molecules and account for material entering and leaving a system. They allow the estimation of contribution from adsorption and removal into the effluent. Mass balance of the cytokines at each time point was calculated as follows:

5 $$M_{{{\text{predialyzer}}}} = \, Q_{{\text{i}}} \times \, C_{{{\text{predialyzer}}}} ; \, Q_{{\text{i}}} = \, Q_{{\text{b}}} \times \, \left( {1 - \frac{{{\text{Hct}}}}{100}} \right)$$

6 $$M_{{{\text{postdialyzer}}}} = \, Q_{{\text{o}}} \times \, C_{{{\text{postdialyzer}}}} ; \, Q_{{\text{o}}} = \, Q_{{\text{i}}} {-} \, Q_{{{\text{uf}}}}$$

7 $$M_{{{\text{total}}}} = \, M_{{{\text{predialyzer}}}} {-} \, M_{{{\text{postdialyzer}}}}$$

8 $$M_{{{\text{df}}}} = \, Q_{{{\text{df}}}} \times \, C_{{{\text{eff}}}}$$

9 $${\text{M}}_{{{\text{ad}}}} = {\text{ M}}_{{{\text{total}}}} {-}{\text{ M}}_{{{\text{df}}}}$$

where *Q*_i_ is inlet plasma flow rate (ml/min); *Q*_b_ is blood flow rate (ml/min); Hct is haematocrit at sampling time; *Q*_o_ is outlet plasma flow rate (ml/min); *Q*_uf_ is ultrafiltration rate (ml/min); *Q*_df_ is dialysate flow rate (ml/min); *M*_predialyzer_ is inlet mass rate (pg/min); *M*_postdialyzer_ is outlet mass rate (pg/min); *M*_total_ is total mass removal rate (pg/min); *M*_df_ is mass removal rate by CVVHD (pg/min); *M*_ad_ is mass removal rate by membrane adsorption (pg/min).

We only included subjects with detectable pre-dialyzer concentrations when analysing the effluent and adsorptive clearance rates and mass balances.

#### Reduction ratios

The reduction ratio (RR) of plasma cytokine concentrations at each time point was calculated as follows [24]:

10 $${\text{RR}} = \frac{{{\text{Cpredialyzer}}\;{\text{timeX}} - {\text{Ctime}}0 }}{{{\text{Ctime0}}}} \times 100$$

where Ctime0 = plasma concentrations of cytokines at baseline before CVVHD initiation.

### Statistical analyses

The Kolmogorov–Smirnov test was performed to test for normal distribution of continuous variables. Normally distributed data were summarised as mean ± standard deviation. Missing data were not imputed. Nonparametric variables were summarised as median with interquartile range. Changes in median levels over time were compared using generalised estimating equations (GEE). Spearman’s correlation was performed to assess the correlation between plasma cytokine concentrations and clearance rates. Linear regression was performed to investigate the link between molecular weight and clearance. A *p* value < 0.05 was considered statistically significant. Data were analysed using Stata 16 (StataCorp, College Station, Texas).

## Results

### Patient characteristics

Thirteen patients were recruited to the study, but one patient was excluded because KRT was not started for clinical reasons. Baseline characteristics, severity scores, clinical and laboratory data at KRT initiation of the remaining 12 patients are presented in Table 1. The median dialysate volume was 2400 mL (IQR 2300–3000), and the median ultrafiltration rate was 40 mL/h (IQR 0–190). Eight patients were discharged alive from the ICU.

**Table 1:** Baseline characteristics of study patients before CVVHD initiation

Parameters	*n* = 12
Baseline data	
Age (median, IQR)	57 (47.8, 75)
Male (n)	8
Source of sepsis (n)	
Lung	5
Abdomen	5
Musculoskeletal system	2
Comorbidities (n)	
Diabetes	3
Hypertension	6
Chronic heart disease	3
Chronic lung disease	1
Cerebrovascular disease	3
Weight (kg)	72.5 (70, 87)
Height (cm)	176 (165, 179)
Parameters on day of enrolment	
Mechanical ventilation (n, %)	9 (75%)
Vasopressor use (n, %)	9 (75%)
APACHE II (median, IQR)	27 (23, 30)
SOFA score (median, IQR)	11 (10, 12)
Urine output (mL/day)	63 (33, 161)
Laboratory data (median, IQR)	
Haemoglobin (g/L)	86 (71, 104)
Urea (mmol/L)	13.1 (11.5, 18)
Creatinine (µmol/L)	287 (150, 438)
C-reactive protein (mg/L)	127 (82, 228)

*IQR* interquartile range, *APACHE* Acute Physiologic and Chronic Health Evaluation II, *SOFA* Sequential Organ Failure Assessment

### Cytokine plasma concentrations

**Fig. 1:**
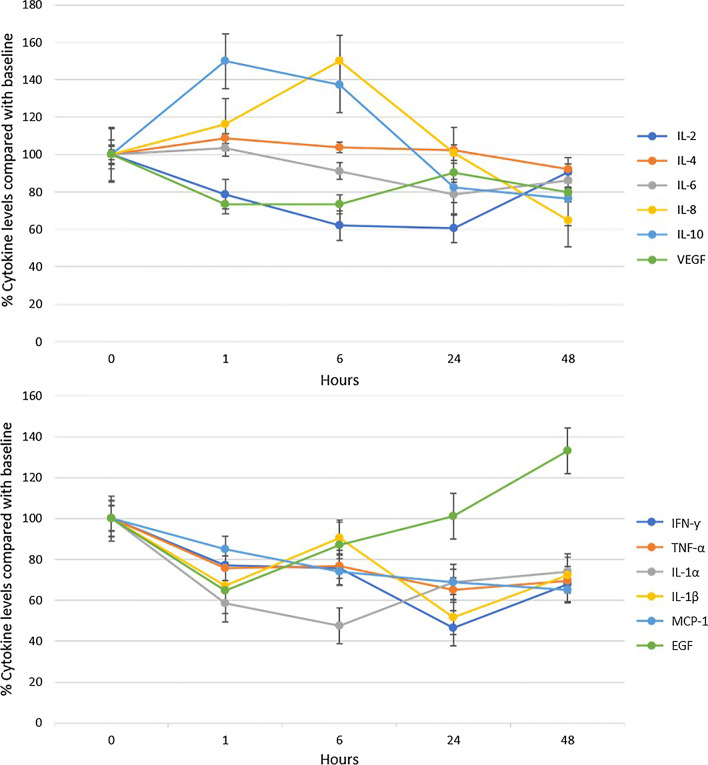
Cytokine plasma concentrations expressed as a percentage of the concentration at time = 0 h. **a** Mean and standard error of interleukin-2 (IL-2), IL-4, IL-6, IL-8, IL-10, vascular endothelial growth factor (VEGF) concentrations. **b** Mean and standard error of interferon-γ (IFN-γ), tumour necrosis factor-α (TNF-α), IL-1α, IL-1β, monocyte chemoattractant protein-1 (MCP-1), epidermal growth factor (EGF)

Median and interquartile range of pre-dialyzer plasma cytokine concentrations at baseline and pre-determined time points are displayed in Table 2. IL-2, EGF, IFN-γ, IL-1β were undetectable in 3, 3, 2, and 1 patient, respectively, throughout the whole study period. The plasma concentrations of all cytokines except EGF significantly decreased over time (*p* < 0.001). Figure 1 demonstrates the pre-filter cytokine concentrations at each time point relative to baseline levels (T0, 100%). At 48 h, the pre-filter cytokine concentrations decreased to 38.98 ± 18.89% for IFN-γ and to 90.57 ± 52.21% for IL-2, corresponding to reduction ratios of -61.02% and -9.43%, respectively. (Additional file 2) In contrast, for EGF, the pre-filter cytokine concentrations at 48 h were 161.74 ± 97.84% higher compared with baseline.

**Table 2:** Pre-filter cytokine concentrations at pre-determined time points

Cytokines (pg/ml)	T0 (*n* = 12)	T1 (*n* = 12)	T6 (*n* = 12)	T24 (*n* = 11)	T48 (*n* = 7)	*p* value*
IL-2	2.68 (0,4.18)	1.21 (0, 2.73)	0 (0, 2.67)	0 (0, 3.32)	0 (0, 4.75)	< 0.001
IL-4	1.30 (1.17, 1.92)	1.4 (1.16, 2.03)	1.36 (1.15, 1.84)	1.19 (0.99, 2.24)	1.19 (0.99,1.94)	< 0.001
IL-6	311.03 (30.92, 1175.13)	84.22 (35.02, 1336.48)	80.64 (30.12, 1273.17)	27.48 (23.33, 548.69)	90.82 (14.4,153.54)	< 0.001
IL-8	83.21 (55.10, 588.14)	74.15 (21.79, 446.88)	88.83 (20.82, 356.09)	60.44 (22.65, 222.59)	37.28 (19.99, 77.22)	< 0.001
IL-10	9.35 (4.38, 25.46)	10.04 (4.35, 27.55)	9.03 (3.96, 19.89)	6.58 (3.32, 9.42)	4.95 (1.54, 6.93)	0.008
VEGF	41.25 (21.78, 64.97)	35.86 (10.46, 59.43)	29.00 (14.9, 35.68)	30.28 (20.77, 39.77)	28.64 (13.93, 38.23)	< 0.001
IFN-γ	1.59 (1.31, 1.92)	1.62 (0,2.76)	1.01 (0, 2.34)	0 (0, 1.89)	0 (0, 1.46)	< 0.001
TNF-α	5.84 (4.55, 18.03)	5.30 (3.2, 13.33)	4.63 (3.44, 13.83)	4.14 (2.85, 9.1)	4.08 (3.14, 4.61)	< 0.001
IL-1α	0.72 (0, 1.59)	0.24 (0, 1.27)	0 (0, 1.07)	0 (0, 1.29)	0.56 (0, 1.33)	< 0.001
IL-1β	1.37 (0.5, 2.79)	1.09 (0, 1.71)	1.19 (0, 1.77)	0 (0, 1.47)	0 (0, 1.64)	< 0.001
MCP-1	571.9 (335.2, 1436.69)	516.75 (311.89, 779.07)	457.74 (275.88, 646.24)	316.63 (201.93, 742.44)	262.63 (116.93, 451.12)	< 0.001
EGF	1.34 (0, 1.49)	0 (0, 1.66)	1.30 (0, 1.62)	1.38 (0, 1.68)	1.76 (0,1.84)	< 0.001

Values expressed as median (interquartile range)

Normal range from literature for baseline cytokine concentrations in sepsis (in pg/mL):

IL-2 = 0 (0–108.5); IL-4 = 8.88 (93.2–202.7); IL-6 = 376–2375; TNF-α = 4.43 to 33; IL-8 = 215 to 1349; IL-1β = 0 to 66.02; IL-10 = 88.55 to 638; IFN-γ = 20.82 to 275 pg/mL; MCP-1 = 454.2 (97.4–22,000); EGF = 35.9 ± 58.03, VEGF = 27.58 to 1082, IL-1α = 0.12 to 0.36 pg/mL [25–34]

*IL* interleukin, *VEGF* vascular endothelial growth factor, *IFN* interferon, *TNF* tumour necrosis factor, *MCP* monocyte chemoattractant protein, *EGF* epidermal growth factor

^*^p value for differences in cytokine concentrations for all time points using generalized estimating equations (GEE)

### Cytokine removal

#### Clearance rates

The adsorption and effluent clearance rates at each time point are shown in Table 3 and Additional file 3. The median time-weighted effluent clearance rates varied from 0 mL/min in 5 cytokines (IL-2, IFN-γ, IL-1α, IL-1β, and EGF) to 33.9 mL/min (MCP-1), 55.4 mL/min (IL-4), and 63.8 mL/min (IL-8). The effluent clearance rates were constant during the 48-h period for most cytokines, except for IL-10 and TNF-α where clearance rates were higher at T48. The median time-weighted adsorption rates ranged from − 64.0 ml/min (IQR − 91.2 to − 43.4) for IL-4 to 9.8 ml/min (IQR − 3.8 to 46.3) for IL-2. Negative values for adsorption were observed in all cytokines at some time points. There were no significant changes in adsorption rates over time. Correlations between serum concentrations and adsorption and effluent clearance are shown in Additional file 4.

**Table 3:** Serial cytokine clearance rates by adsorption and diffusion at 1, 6, 24, and 48 h after continuous veno-venous haemodialysis initiation and time-weighted mean clearance rates

Cytokines	T1	T6	T24	T48	Time-weighted mean
IL-2					
Adsorption (*n* = 6)	3.0 (− 21.9, 72)	18.5 (11.5, 20.6)	18.8 (− 3.6, 75)	6.4 (− 4.0, 9.8)	9.8 (− 3.8, 46.3)
Effluent (*n* = 6)	0 (0, 24.2)	0 (0, 0)	0 (0, 0)	0 (0, 0)	0 (0, 0)
IL-4					
Adsorption (*n* = 12)	− 54.2 (− 107.7, − 38.7)	− 61.0 (− 75.3, − 44.2)	− 78.1 (− 91.7, − 46.9)	− 61.3 (− 67.8, − 50.8)	− 64.0 (− 91.2, − 43.4)
Effluent (*n* = 12)	55.2 (38.9, 69.0)	55.9 (50, 66.0)	59.3 (52.1, 72.2)	68.9 (43.2, 83.9)	55.4 (51.3, 69.9)
IL-6					
Adsorption (*n* = 12)	− 15.3 (− 21.6, − 3.1)	− 4.9 (− 11.5, 4.8)	− 13.3 (− 20.8, − 1.3)	− 10.6 (− 13.1, − 5.7)	− 11.5 (− 18.1, 0.9)
Effluent (*n* = 12)	10.2 (6.1, 17.3)	9.0 (8.1, 10.6)	5.6 (2.4, 20.5)	2.2 (0, 6.8)	7.8 (4.1, 15.7)
IL-8					
Adsorption (*n* = 12)	− 44.5 (− 66, − 23)	− 42.3 (− 224.1, − 19)	− 50 (− 243.3, − 28.8)	− 61.8 (− 100.9, − 2.9)	− 48.9 (− 144.7, − 19.0)
Effluent (*n* = 12)	37.7 (26.4, 47.8)	49.0 (40.8, 87.5)	62.7 (24.2, 116.3)	32.7 (16.2, 103.1)	63.8 (34.7, 93.6)
IL-10					
Adsorption (*n* = 12)	− 5.9 (− 16.7, 1.6)	3.5 (− 10.0, 7.1)	− 6.6 (− 10.1, 8.2)	− 17.2 (− 25.7, − 8.5)	− 7.1 (− 10.7, 16.9)
Effluent (*n* = 12)	0.5 (0, 10.0)	0 (0, 1.8)	0 (0, 1.8)	9.6 (4.5, 21.3)	2.2 (0.3, 5.5)
VEGF					
Adsorption (*n* = 9)	− 2.7 (− 22.8, 11.2)	− 2.6 (− 6.9, 10.6)	4.0 (− 7.4, 17.2)	37.7 (0.5, 100)	4.0 (− 7.4, 16.9)
Effluent (*n* = 10)	5.0 (0, 17.6)	0 (0, 20.2)	0 (0, 0)	0 (0, 16.8)	3.4 (0, 19.2)
IFN-γ					
Adsorption (*n* = 7)	0 (− 20.0, 24.1)	7.9 (4.4, 12.3)	16.6 (− 20.4, 5.7)	− 16.1 (− 20.9, − 4.5)	− 2.3 (− 20.3, 11.2)
Effluent (*n* = 7)	0 (0, 0)	0 (0, 0)	0 (0, 0)	0 (0, 0)	0 (0, 0)
TNF-α					
Adsorption (*n* = 12)	− 5.3 (− 10.6, 3.5)	− 7.0 (− 12.3, 4.1)	− 22.8 (− 38.8, − 13.4)	− 19.3 (− 23.4, − 0.6)	− 10.6 (− 23.3, 4.1)
Effluent (*n* = 12)	11.6 (0, 17.0)	19.0 (13.9, 30.7)	26.5 (13.5, 33.2)	22.7 (18.5, 28.8)	19.0 (9.0, 28.4)
IL-1α					
Adsorption (*n* = 6)	− 7.2 (− 13.1, 69)	− 5.6 (− 30.2, 5.0)	− 26.1 (− 38.9, − 7.7)	− 11.4 (− 46.1, 48.1)	− 10.2 (− 23.2, 4.1)
Effluent (*n* = 6)	0 (0, 0)	0 (0, 35.0)	0 (0, 15.2)	0 (0, 9.3)	0 (0, 8.6)
IL-1β					
Adsorption (*n* = 7)	− 12.2 (− 62.3, 0)	22.5 (− 3.6, 56)	− 21.1 (− 46.0, − 10.2)	− 9.2^a^	− 8.6 (− 21.1, 3.1)
Effluent (*n* = 7)	0 (0, 29.0)	0 (0, 20.0)	30.0 (0, 61.4)	0^a^	0 (0, 31.8)
MCP-1					
Adsorption (*n* = 12)	− 31.8 (− 19.9, − 13.4)	− 23.1 (− 25.4, − 17.8)	− 30.5 (− 35.7, − 10.9)	− 22.9 (− 40.5, − 4.8)	− 24.9 (− 35.7, − 10.9)
Effluent (*n* = 12)	33.5 (29.7, 46.6)	38.2 (35.0, 43.0)	31.3 (18.0, 50)	32.9 (24.5, 46.6)	33.9 (21.6, 43.6)
EGF					
Adsorption (*n* = 5)	0.9 (0.7, 1.7)	8.2 (0, 15.1)	− 7.3 (− 14.8, − 4.8)	− 0.2 (− 43.0, 3.7)	0 (− 12, 3.4)
Effluent (*n* = 7)	0 (0, 0)	0 (0, 0)	0 (0, 0)	0 (0, 0)	0 (0, 0)

Values expressed as median (interquartile range)

*IL* interleukin, *VEGF* vascular endothelial growth factor, *IFN* interferon, *TNF* tumour necrosis factor, *MCP* monocyte chemoattractant protein, *EGF* epidermal growth factor, T1 = 1 h, T6 = 6 h, T24 = 24 h, and T48 = 48 h

^a^Only one observation

#### Mass balance

**Fig. 2:**
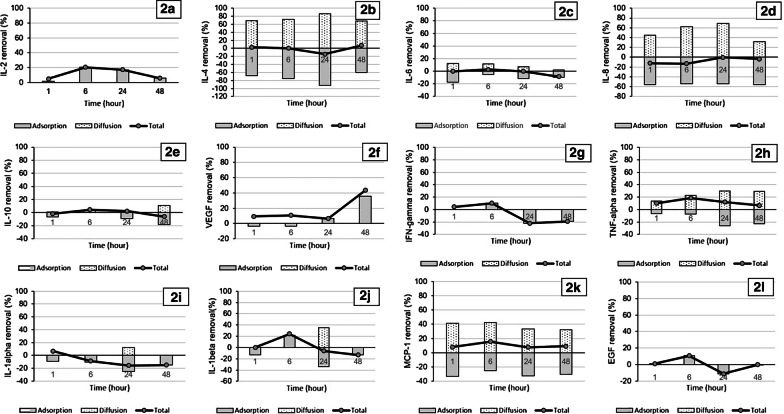
The median levels of total amount of cytokine moved (*M*_t_), expressed as a percentage of the inlet mass rate (%*M*_t_/*M*_i_) at *t* = 1, 6, 24, and 48 h after continuous veno-venous haemodialysis initiation (connected line). For each time point, the relative proportions of adsorption (%*M*_ad_/*M*_i_, gray bars) and haemofilter clearance (%*M*_df_/*M*_i_, dotted bars) are shown. **a** IL-2, **b** IL-4, **c** IL-6, **d** IL-8, **e** IL-10, **f** VEGF, **g** IFN-γ, **h** TNF-α, **i** IL-1α, **j** IL-1β, **k** MCP-1, **l** EGF

The total mass transfer (*M*_total_) and mass balance via adsorption (*M*_ad_) and CVVHD (*M*_df_) are demonstrated in Additional file 5. Total mass transfer and the contributions from *M*_df_ and *M*_ad_ were calculated in percentage of *M*_predialyzer_ and shown in Fig. 2. There was marked heterogeneity in the proportion of haemofilter clearance and adsorption for all cytokines over time. CVVHD (%*M*_df_/*M*_predialyzer_) contributed more to cytokine removal than adsorption (%*M*_ad_/*M*_predialyzer_) for IL-4, TNF-α, and MCP-1. At 1 h after CVVHD initiation, total removal (%*M*_total_/*M*_predialyzer_) ranged from − 12.24% (IL-8) to 10.27% (TNF-α). The total cytokine removal rates remained stable over the observation period except for VEGF which rose due to increased adsorption. At 48 h, %*M*_total_/*M*_predialyzer_ varied from − 19.06% (IFN-γ) to 43.54% (VEGF).

## Discussion

This is the first study which investigated the transport characteristics of 12 molecules across the EMiC2 membrane. The key findings were: first that the plasma concentrations of all molecules declined over 48 h, except for EGF. Second, the effluent clearance rates were low for most cytokines, except for IL-4, IL-8, and MCP-1. Third, minimal or negative adsorption was observed for all cytokines. Finally, the total removal rates and contributions from CVVHD and adsorption were heterogeneous but were mostly low to moderate.

Extracorporeal blood purification to attenuate the effects of pro-inflammatory and anti-inflammatory mediators in sepsis remains a controversial issue. Although some studies showed potentially promising results and a reduction in cytokine concentrations in plasma with particular extracorporeal techniques, it is not always clear whether these declines in cytokine levels are related to the filter, or simply a reflection of the dynamic nature of sepsis [10]. Baseline cytokine concentrations of our patient cohort were similar or lower than previous reports in the literature [32–41]. This possibly reflects the heterogeneity in phenotypes and severity of sepsis and also underlines the complexity of cytokine profile interpretation in this setting. Whilst some cytokines are pro-inflammatory and associated with poor outcomes [42], others have a potentially beneficial role. For instance, EGF represents tissue recovery or regeneration after injury and is associated with cellular proliferation and survival in sepsis [43].

Previous studies explored cytokine changes and cytokine removal by the EMiC2 filter, but none have fully investigated the various mechanisms of cytokine clearance in critically ill patients with sepsis [25–28]. Despite reported decent removal of IL-6, IL-8, IL-1β, and TNF-α (MW 8.4–25 kDa) in-vitro, the effluent clearance rates varied markedly in humans [26, 27, 29]. Several factors might affect in vivo clearance, e.g. duration of blood contact, binding to protein or plasma components, dialysate rate or ultrafiltration rate, molecular weight, serum concentration, or sampling time after filter installation. In a previous study using a HCO membrane, a decline in plasma IL-1ra and IL-6 was observed in patients with high baseline concentrations [11]. This corresponds with our results showing that serum IL-2, IL-6, and IL-1β concentrations were positively correlated with effluent clearance rates. We also noted that the effluent clearance rates remained constant over time which may be explained by the use of citrate-based anticoagulation [44]. Citrate can prolong filter life by reducing both filter clotting (i.e. thrombosis within the lumen of the filter) and clogging (i.e. formation of protein layer which progressively obliterates the membrane pores) [45, 46].

The contribution of adsorption to total cytokine removal with EMiC2 filters was minimal. We noted a small degree of adsorption of IL-2 and VEGF which contributed to total mass removal. However, “negative adsorption” was also observed for all cytokines consistent with similar reports in the literature [15, 28, 31]. The mechanisms for this “de-sorption” phenomenon are unclear and might be explained by effects of haemoconcentration on the outflow side from fluid removal albeit in a significantly smaller degree than in CVVH mode, release of previously bound cytokines from cells [47], activation of the inflammatory system through reverse diffusion [28], the ‘back-filtration/back-diffusion’ phenomenon including the movement of molecules from a higher concentration in the dialysate to a lower concentration in the blood at the distal end of the hollow fibres in high-flux membranes [48], cytokine induction from dialyzer bio-incompatibility [49], activation or deactivation by enzymes after sampling, or sampling errors.

We found that the changes in cytokine concentrations seen in plasma were discordant with the extent of removal by clearance and adsorption. Despite a decline in serum concentrations, we found low total mass removal rates across the filter for most cytokines. The clearance rates were highest for IL-4, IL-8, and MCP-1, but their plasma reduction ratios varied significantly from − 13.46 to − 59.94%. Although VEGF showed the highest total mass removal at 48 h, the reduction ratio was − 34.88% which was lower than others.

Some of our findings are compatible with data in the literature but not all. For instance, a previous study demonstrated higher IL-6 and IL-8 clearance by the EMiC2 filter than the standard membrane, but showed no significant impact on plasma cytokine concentrations [26]. Another study showed comparable IL-6 clearance between the EMiC2 and high-flux membrane [27]. Similarly, studies using the EMiC2 or HCO membranes reported no changes in plasma concentrations despite detectable clearance in the ultrafiltrate [11, 15, 28, 50]. These results in the literature, together with our findings highlight that a rise or fall in serum concentrations during KRT might be related to factors other than extracorporeal removal, e.g. changes in cytokine production, endogenous clearance, intradialytic cytokine release, general improvement of the underlying disease, or response to treatment [51]. It should also be noted that cytokine half-lives are extremely short. (Additional file 6) Thus, their endogenous metabolism might be more rapid than clearance during extracorporeal therapy. In general, the kidneys contribute to 15–20% of cytokine metabolism [52]. Our study showed that CVVHD using the EMiC2 filter contributed little to total clearance of most cytokines. This suggests that extracorporeal removal does not substitute renal clearance, similar to lactate removal [53]. In addition, the interaction of soluble forms and their endogenous modulators (receptors or antagonists) indicates different states of immune activation. Thus, the measurement of plasma cytokine concentrations in isolation might not reflect the true immune status or indicate the impact of extracorporeal removal on the dynamics of that particular cytokine system [54].

This is the first study to investigate a comprehensive panel of molecules which are representative of pro- and anti-inflammatory cytokines in real clinical settings. Adsorption and diffusive clearances were evaluated extensively by determination of their clearance rates, and mass balances across the membrane over a 48-h period. However, some limitations need to be acknowledged. First, the objective of this pilot study was to investigate the mechanistic impact of using the EMiC2 filter. It was not powered to assess an association with clinical outcomes. Second, we did not intend to compare the EMiC2 filter with other filters. Therefore, there was no control group. However, this is an exploratory study to characterize the transport characteristics of cytokines when using the EMiC2 filter. This investigation is essential before proceeding to larger clinical studies investigating the role of blood purification with the EMiC2 filter as an adjunctive therapy in sepsis. Third, we selected 12 different molecules but did not measure all potential pro- and anti-inflammatory cytokines. We acknowledge that our conclusions only apply to the cytokines measured and that it is possible that other cytokines or medium-sized molecules are removed at higher quantities when using the EMiC2 filter. Fourth, the cohort of included patients was heterogenous and there were some patients with undetectable cytokine concentrations throughout the whole study period. Given the mechanistic nature of this project, we only included subjects with detectable pre-dialyzer concentrations when analysing the effluent and adsorptive clearance rates and mass balances. Although there was minimal correlation between initial cytokine concentrations and clearance rates, we are unable to comment on whether cytokine removal in patients with different cytokine concentrations, including cytokine storm/septic shock would have different clinical effects. We acknowledge that cytokines may have different effects independent of the concentration, including paracrine and endocrine actions [55]. Finally, our aim was to describe clearance and adsorption of cytokines during CVVHD with an EMiC2 filter. Although we showed that only small amounts of cytokines were actually removed, we acknowledge that we cannot exclude any immunomodulatory effects [56, 57].

## Conclusion

Our study has shown that in patients with sepsis and acute kidney injury requiring KRT with the EMiC2 filter, clearance of cytokines by CVVHD was modest and adsorption was minor. We observed a decline in serum concentrations of most cytokines during the study period but were unable to detect an obvious correlation between serum concentration and cytokine clearance. The results suggest that mechanisms other than extracorporeal removal contribute to changes in plasma cytokine concentrations. Further work to determine the role of the EMiC2 filter in clinical practice is required.

## Supplementary Information

**Additional file 1.** Sites of sampling from the CVVHD circuit.

**Additional file 2.** Reduction ratio of cytokine concentrations at *t* = 1 (*n* = 12), 6 (*n* = 12), 24 (*n* = 11), and 48 (*n* = 7) hours compared with baseline pre-filter concentrations (%)

**Additional file 3.** Clearance rates (mL/min) of cytokines by adsorption (pink bars) and effluent (blue bars) over time visualized as box and whisker plots (horizontal bars indicate median values).

**Additional file 4.** Spearman's correlation between serum levels and clearances by adsorption and effluent.

**Additional file 5.** Mass balances for all cytokines (pg/min): Mass removal rate by adsorption (Mad), mass removal rate by dialysis (Mdf), and total mass removal rate (Mt).

**Additional file 6.** Cytokine half-lives as reported in literature.

## Data Availability

The datasets used and/or analysed during the current study are available from the corresponding author on reasonable request.

## Abbreviations

AKI: Acute kidney injury
APACHE: Acute Physiologic and Chronic Health Evaluation
BPT: Blood purification therapy
Ceff: Effluent concentration
Cleff: Effluent clearance
Clmean: Time weighted average clearance
Cltotal: Total clearance
Cpredialyzer: Predialyzer serum concentration
Cpostdialyzer: Postdialyzer serum concentration
CKRT: Continuous kidney replacement therapy
CVVHD: Continuous veno-venous haemodialysis
CVVHDF: Continuous veno-venous haemodiafiltration
EGF: Epidermal growth factor
HCO: High cut-off
Hct: Haematocrit
HVHF: High-volume haemofiltration
IFN: Interferon
ICU: Intensive care unit
IL: Interleukin
IQR: Interquartile range
K_ad_: Adsorptive clearance
kDa: Kilodalton
KRT: Kidney replacement therapy
M_ad_: Mass removal by adsorption
M_df_: Mass removal by dialysis
M_predialyzer_: Predialyzer mass balance
M_postdialyzer_: Postdialyzer mass balance
M_t_: Total mass removal rate
MCO: Medium cut-off
MCP: Monocyte chemoattractant protein
PMMA: Polymethyl methacrylate
PS: Polysulfone
Q_b_: Blood flow rate
Q_i_: Predialyzer plasma flow rate
Q_df_: Dialysate flow rate
Q_o_: Postdialyzer plasma flow rate
Q_uf_: Ultrafiltration rate
RR: Reduction ratio
SOFA: Sequential Organ Failure Assessment
sTNF-Rs: Soluble tumour necrosis factor-receptors
TNF: Tumour necrosis factor
VEGF: Vascular endothelial growth factor
