# Supplementary material for: Clearance of inflammatory cytokines in patients with septic acute kidney injury during renal replacement therapy using the EMiC2 filter (Clic-AKI study)
Source: Crit Care. 2021 Jan 28;25:39. doi: 10.1186/s13054-021-03476-x (PMC7845048; doi:10.1186/s13054-021-03476-x)
Supplement: Supplementary file 1 — Additional file 1. Sites of sampling from the CVVHD circuit. [file 13054_2021_3476_MOESM1_ESM.docx]

**Additional file 1** Sites of sampling from the CVVHD circuit

(A) Pre-dialyzer sampling port, (B) Post-dialyzer sampling port, (C) Effluent fluid sampling port


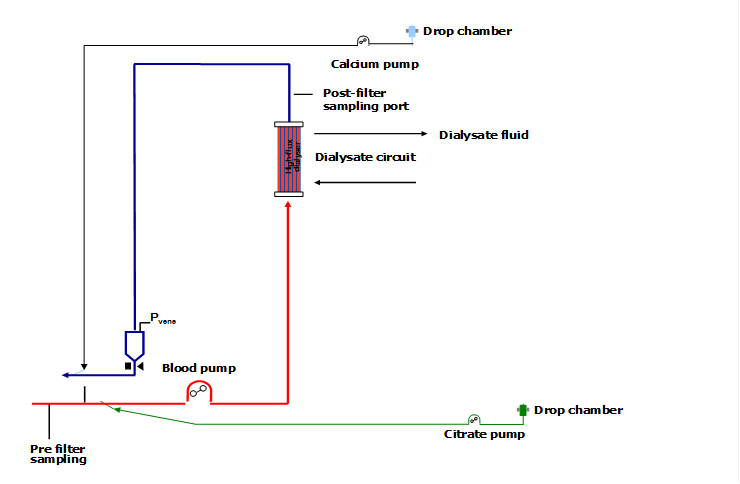


(A) Pre-dialyzer sampling port

(B) Post-dialyzer sampling port

(C) Effluent fluid sampling port
