# Supplementary material for: Clearance of inflammatory cytokines in patients with septic acute kidney injury during renal replacement therapy using the EMiC2 filter (Clic-AKI study)
Source: Crit Care. 2021 Jan 28;25:39. doi: 10.1186/s13054-021-03476-x (PMC7845048; doi:10.1186/s13054-021-03476-x)
Supplement: Supplementary file 2 — Additional file 2. Reduction ratio of cytokine concentrations at t = 1 (n = 12), 6 (n = 12), 24 (n = 11), and 48 (n = 7) hours compared with baseline pre-filter concentrations (%) [file 13054_2021_3476_MOESM2_ESM.docx]

**Additional file 2** Reduction ratio of cytokine concentrations at t = 1 (n=12), 6 (n=12), 24 (n=11), and 48 (n=7) hours compared with baseline pre-filter concentrations (%)

| **Cytokines** | **T0-T1 (%)** | **T0-T6 (%)** | **T0-T24 (%)** | **T0-T48 (%)** |
| --- | --- | --- | --- | --- |
| IL-2 | -21.15  (-62.57, 20.27) | -38.01  (-93.36, 17.34) | -39.44  (-100.89, 22.02) | -9.43  (-143.64, 124.78) |
| IL-4 | 7.80  (-13.75, 29.35) | 3.03  (-17.13, 23.19) | 1.76  (-23.18, 26.71) | -13.46  (-29.25, 2.34) |
| IL-6 | 39.06  (-30.24, 108.36) | 26.68  (-50, 103.36) | -11.45  (-61.09, 38.19) | -24.50  (-146.34, 97.34) |
| IL-8 | 14.66  (-91.26, 120.59) | 48.98  (-133.75, 231.70) | 0.09  (-78.78, 78.97) | -58.93  (-84.20, -33.67) |
| IL-10 | 72.12  (-83.07, 227.32) | 56.92  (-84.44, 198.29) | 2.58  (-70.02, 75.17) | -43.04  (-90.02, 3.94) |
| VEGF | -26.47  (-59.58, 6.64) | -28.98  (-56.01, -1.94) | -14.42  (-53.64, 24.80) | -34.88  (-70.67, 0.91) |
| IFN-ƴ | -14.43  (-60.85, 31.99) | -15.74  (-89.63, 58.15) | -54.46  (-99.65, -9.27) | -61.02  (-107.25, -14.79) |
| TNF-α | -21.70  (-45.31, 1.91) | -20.88  (-55.25, 13.50) | -36.93  (-63.39, -10.47) | -47.18  (-74.91, -19.46) |
| IL-1α | -41.63  (-97.79, 14.54) | -52.55  (-97.43, -7.67) | -35.69  (-100.01, 28.64) | -50.98  (-106.86, 4.90) |
| IL-1β | -27.17  (-78.40, 24.06) | -3.73  (-84.92, 77.45) | -46.82  (-95.60, 1.97) | -54.04  (-128.93, 20.85) |
| MCP-1 | -8.73  (-28.98, 11.52) | -20.51  (-42.61, 1.59) | -30.48  (-49.41, -11.55) | -59.94  (-75.33, -44.55) |
| EGF | -42.42  (-86.47, 1.63) | -22.63  (-58.92, 13.66) | -0.78  (-32.63, 31.08) | 61.74  (-209.90, 333.38) |

Notes: Values expressed as median (interquartile range)

**Abbreviations:** IL, interleukin; VEGF, vascular endothelial growth factor; IFN, interferon; TNF, tumor necrosis factor; MCP, monocyte chemoattractant protein; EGF, epidermal growth factor
