# Supplementary material for: Clearance of inflammatory cytokines in patients with septic acute kidney injury during renal replacement therapy using the EMiC2 filter (Clic-AKI study)
Source: Crit Care. 2021 Jan 28;25:39. doi: 10.1186/s13054-021-03476-x (PMC7845048; doi:10.1186/s13054-021-03476-x)
Supplement: Supplementary file 3 — Additional file 3. Clearance rates (mL/min) of cytokines by adsorption (pink bars) and effluent (blue bars) over time visualized as box and whisker plots (horizontal bars indicate median values). [file 13054_2021_3476_MOESM3_ESM.docx]

**Additional file 3** Clearance rates (mL/min) of cytokines by adsorption (pink bars) and effluent (blue bars) over time visualized as box and whisker plots (horizontal bars indicate median values).

Comparison between clearance rates at each time point and t = 1 hour were indicated by the asterisks (*) for statistical significance (P < 0.05).


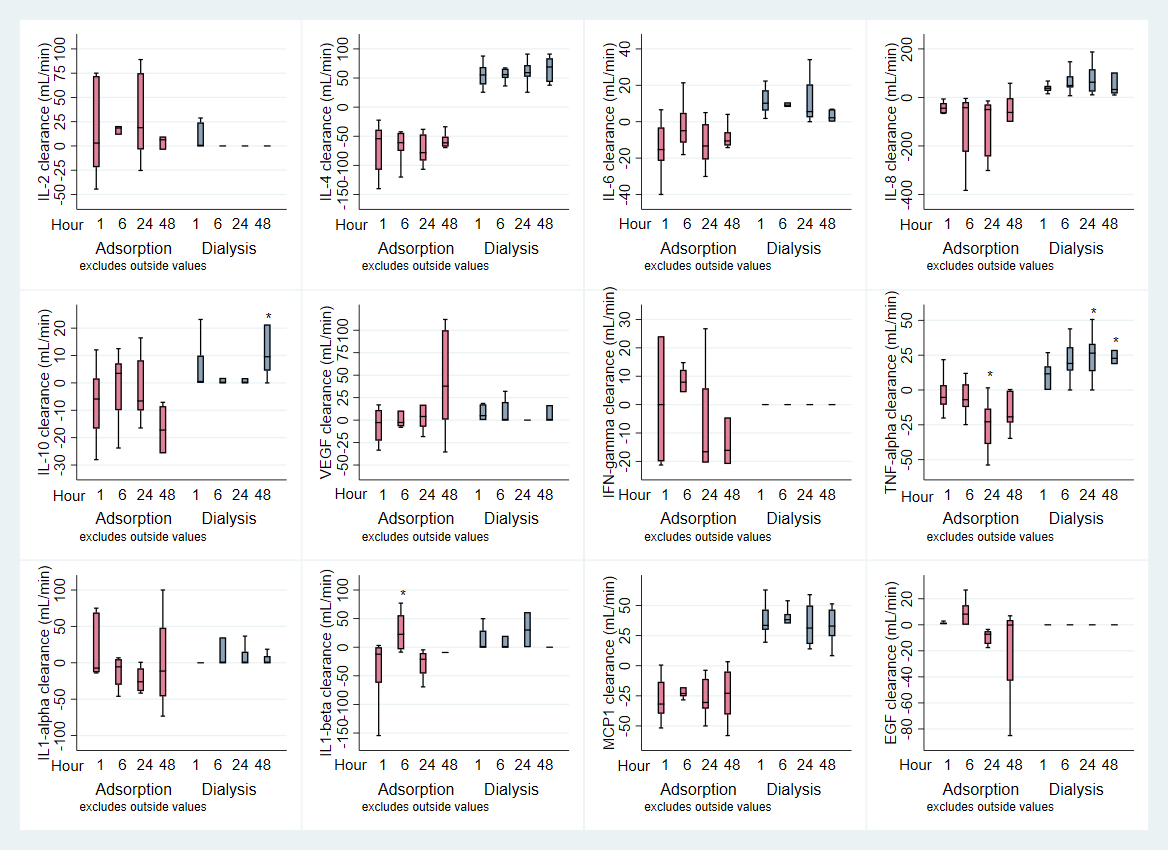


**Abbreviations:** IL, interleukin; VEGF, vascular endothelial growth factor; IFN, interferon; TNF, tumor necrosis factor; MCP, monocyte chemoattractant protein; EGF, epidermal growth factor
