# Supplementary material for: Clearance of inflammatory cytokines in patients with septic acute kidney injury during renal replacement therapy using the EMiC2 filter (Clic-AKI study)
Source: Crit Care. 2021 Jan 28;25:39. doi: 10.1186/s13054-021-03476-x (PMC7845048; doi:10.1186/s13054-021-03476-x)
Supplement: Supplementary file 4 — Additional file 4. Spearman's correlation between serum levels and clearances by adsorption and effluent. [file 13054_2021_3476_MOESM4_ESM.docx]

**Additional file 4** Spearman's correlation between serum levels and clearances by adsorption and effluent

| **Cytokines** | **Adsorption** | **P value** | **Effluent** | **P value** |
| --- | --- | --- | --- | --- |
| IL-2 | -0.52 | 0.03 | 0.35 | 0.17 |
| IL-4 | 0.52 | 0.0008 | -0.71 | <0.001 |
| IL-6 | -0.53 | 0.0005 | 0.54 | 0.0004 |
| IL-8 | 0.18 | 0.28 | -0.09 | 0.57 |
| IL-10 | 0.29 | 0.01 | -0.15 | 0.37 |
| VEGF | 0.08 | 0.65 | 0.22 | 0.20 |
| IFN-ƴ | 0.12 | 0.60 | 0.22 | 0.35 |
| TNF-α | 0.12 | 0.45 | 0.23 | 0.16 |
| IL-1α | -0.56 | 0.02 | 0.28 | 0.26 |
| IL-1β | -0.10 | 0.67 | 0.50 | 0.03 |
| MCP-1 | -0.24 | 0.14 | 0.008 | 0.96 |
| EGF | 0.06 | 0.77 | -0.32 | 0.13 |

**Abbreviations:** IL, interleukin; VEGF, vascular endothelial growth factor; IFN, interferon; TNF, tumor necrosis factor; MCP, monocyte chemoattractant protein; EGF, epidermal growth factor
