# Supplementary material for: Clearance of inflammatory cytokines in patients with septic acute kidney injury during renal replacement therapy using the EMiC2 filter (Clic-AKI study)
Source: Crit Care. 2021 Jan 28;25:39. doi: 10.1186/s13054-021-03476-x (PMC7845048; doi:10.1186/s13054-021-03476-x)
Supplement: Supplementary file 5 — Additional file 5. Mass balances for all cytokines (pg/min): Mass removal rate by adsorption (Mad), mass removal rate by dialysis (Mdf), and total mass removal rate (Mt). [file 13054_2021_3476_MOESM5_ESM.docx]

**Additional file 5** Mass balances for all cytokines (pg/min): Mass removal rate by adsorption (Mad), mass removal rate by dialysis (Mdf), and total mass removal rate (Mt)

| **Cytokines** | **Mass removal rate (pg/min)** | **T1** | **T6** | **T24** | **T48** |
| --- | --- | --- | --- | --- | --- |
| IL-2  (n=6) | Adsorption | 8.7  (-145.7, 174.2) | 54.0  (43.7, 109.1) | 55  (-23.9, 224.3) | 17.3  (-49.4, 46.4) |
|  | Dialysis | 0 (0,0) | 0 (0, 0) | 0 (0, 0) | 0 (0, 0) |
|  | Total | 18.4  (-6.2, 174.2) | 48.8  (20.0, 81.5) | 55  (-23.9, 224.3) | 17.3  (-49.4, 46.4) |
| IL-4  (n=12) | Adsorption | -82.3  (-139.7, -60.7) | -78.5  (-83.9, -73.9) | -92.7  (-109.4, -84.3) | -68.4  (-82.7, -65.9) |
|  | Dialysis | 83.2  (71.8, 94.0) | 87.2  (71, 100.2) | 90.2  (63.1, 103.3) | 84.2  (77.6, 97.5) |
|  | Total | 4.7  (-29.9, 22.9) | -0.9  (-5.9, 11.6) | -15  (-32.8, 2.9) | 5.6  (-6.1, 14.7) |
| IL-6  (n=12) | Adsorption | -970.3  (-19423, -92.8) | -50.2  (-1694, 182) | -298.1  (-11390.8, -31.7) | -672.3  (-1380, -125.6) |
|  | Dialysis | 616.0  (257.7, 19227.5) | 530.6  (60.2, 6608) | 346.5 (69.9, 1742.6) | 224.6  (0, 266.2) |
|  | Total | 0  (-388.5, 469.6) | 219.3  (-3.9, 944) | 0  (-933.4, 228.5) | -684.9  (-1009.9, 5.6) |
| IL-8  (n=12) | Adsorption | -4810.2  (-12008, -1565) | -5352.1  (-30014.1, -654.6) | -5129.8  (-26834, -1061) | -1856.8  (-3903.6, -221) |
|  | Dialysis | 3255.3 (721.3, 12400.5) | 13256.8 (690.4, 16850.8) | 3490  (470.7, 17956.9) | 1763.5  (326, 3149.8) |
|  | Total | -345.6  (-2621.7, 864.8) | -128.5  (-3322.9, 3990.8) | -1.7  (-2187, 240) | 38.3  (-1373, 558.4) |
| IL-10  (n=12) | Adsorption | -34.8  (-145.5, 45.5) | 28.8  (-12.9, 71.7) | -12.3  (-95.6, 19.3) | -54.5  (-95.2, -37.8) |
|  | Dialysis | 19  (0, 75.9) | 0  (0, 47.3) | 0  (0, 43.2) | 35.5  (32.6, 66.3) |
|  | Total | -9.6  (-94.9, 114.2) | 24.2  (-14.6, 70) | 7.1  (-95.6, 51) | -19.3  (-53.8, -13.0) |
| VEGF  (n=9) | Adsorption | -155.8  (-875.6, 371.3) | -35.3  (-174.8, 347.6) | 122.4  (-129.8, 1672.7) | 930.5  (16.3, 1615) |
|  | Dialysis | 0 (0, 610.2) | 0 (0, 610) | 0 (0, 0) | 0 (0, 551.5) |
|  | Total | 371.3  (-580.9, 748.1) | 202  (-35.3, 347.9) | 122.5  (-44.3, 1672.7) | 1068.6 (85.5, 1615.0) |
| IFN-ƴ  (n=12) | Adsorption | 0 (-38.9, 11.1) | 0 (0, 24.6) | 0 (-38.6, 0) | -3.6 (-23.5, 0) |
|  | Dialysis | 0 (0, 0) | 0 (0, 0) | 0 (0, 0) | 0 (0, 0) |
|  | Total | 0 (0, 15.4) | 0 (0, 26.9) | 0 (-38.6, 0) | 0 (-23.5, 0) |
| TNF-α  (n=12) | Adsorption | -19.6  (-69.6, 33.6) | -25.2  (-135.9, 27.8) | -109.4  (-207.5, -39.8) | -67.7  (-109.0, -1.4) |
|  | Dialysis | 63.3  (0, 164.4) | 89.4  (54.7, 208.4) | 102.9  (57.9, 226.8) | 87.2  (79.3, 109.0) |
|  | Total | 78.2  (-5.3, 137.3) | 56.0  (1.5, 266.4) | 40  (-151.8, 86.5) | 43.1  (-1.4, 57.6) |
| IL-1α  (n=6) | Adsorption | -12.7  (-21.3, 32.4) | -5.9  (-75, 6.2) | -48.9  (-85.2, -21.7) | -13.4  (-56.8, 22.7) |
|  | Dialysis | 0 (0, 0) | 0 (0, 0) | 0 (0, 23.3) | 0 (0,0) |
|  | Total | 13.0  (-21.3, 40.3) | 6.1  (-17.9, 35.8) | -14.9  (-73.5, 23.8) | -13.4  (-40.2, 22.7) |
| IL-1β  (n=7) | Adsorption | -73.1  (-102.1, 0) | 36.1  (-9.0, 77.8) | -44.3  (-56.2, -12.3) | -19.7* |
|  | Dialysis | 0 (0, 43.5) | 0 (0, 0) | 0 (0, 51.0) | 0 (0, 0) |
|  | Total | 0  (-15.1, 38.3) | 77.8  (27.7, 115.1) | -6.8  (-12.3, -5.2) | 12.9  (-19.7, 45.6) |
| MCP-1  (n=12) | Adsorption | -12760.7  (-20902, -6595) | -8610.1  (-14341, -6946.3) | -6161.9  (-19019.5, -2951) | -5894.2  (-13309, -493) |
|  | Dialysis | 17960 (12613, 23547) | 15053 (11945, 21341.2) | 9507.2  (6255.7, 16095) | 9416.6 (2561, 12709) |
|  | Total | 2608.3  (0, 8393) | 4985.3 (2009.8, 6719.9) | 1905.8  (0, 3304.5) | 1598.1  (-3683.4, 2948) |
| EGF  (n=5) | Adsorption | 2.2 (1.4, 3) | 15.4 (0, 23.4) | -14.2 (-23.2, -7.9) | 0.9 (-79.0, 7.7) |
|  | Dialysis | 0 (0, 0) | 0 (0, 0) | 0 (0, 0) | 0 (0, 0) |
|  | Total | 2.2 (1.4, 3) | 8.4 (-2.8, 23.4) | -14.2 (-23.2, -7.9) | 0.9 (-79.0, 7.7) |

*only one observation

Notes: Values expressed as median (interquartile range)

**Abbreviations:** IL, interleukin; VEGF, vascular endothelial growth factor; IFN, interferon; TNF, tumor necrosis factor; MCP, monocyte chemoattractant protein; EGF, epidermal growth factor
