# Supplementary material for: Clearance of inflammatory cytokines in patients with septic acute kidney injury during renal replacement therapy using the EMiC2 filter (Clic-AKI study)
Source: Crit Care. 2021 Jan 28;25:39. doi: 10.1186/s13054-021-03476-x (PMC7845048; doi:10.1186/s13054-021-03476-x)
Supplement: Supplementary file 6 — Additional file 6. Cytokine half-lives as reported in literature. [file 13054_2021_3476_MOESM6_ESM.docx]

**Additional file 6** Cytokine half-lives as reported in literature

| **Cytokine** | **Half-life** | **References** |
| --- | --- | --- |
| IL-2 | 5-7 minutes | Lotze MT, Frana LW, Sharrow SO et al. In vivo administration of purified human interleukin 2. I. Half-life and immunologic effects of the Jurkat cell line-derived interleukin 2. J Immunol. 1985;134(1):157-66. |
| IL-4 | 19 minutes | Conlon PJ, Tyler S, Grabstein KH, Morrissey P. Interleukin-4 (B-cell stimulatory factor-1) augments the in vivo generation of cytotoxic cells in immunosuppressed animals. Biotechnol Ther. 1989-1990;1(1):31-41. |
| IL-6 | 10 minutes | Oda S, Hirasawa H, Shiga H et al. Sequential measurement of IL-6 blood levels in patients with systemic inflammatory response syndrome (SIRS)/sepsis. Cytokine. 2005;29:169-75. |
| IL-8 | 4 hours | Orlikowsky TW, Neunhoeffer F, Goelz R et al. Evaluation of IL-8-concentrations in plasma and lysed EDTA-blood in healthy neonates and those with suspected early onset bacterial infection. Pediatr Res. 2004;56(5):804-9. |
| IL-10 | 2-7 hours | Huhn RD, Radwanski E, Gallo J et al. Pharmacodynamics of subcutaneous recombinant human interleukin-10 in healthy volunteers. Clin Pharmacol Ther. 1997;62(2):171-80. |
| IL-1alpha | 30 minutes | Kondo S, Sauder DN, Kono T et al. Differential modulation of interleukin-1 alpha (IL-1 alpha) and interleukin-1 beta (IL-1 beta) in human epidermal keratinocytes by UVB. Exp Dermatol. 1994;3(1):29-39. |
| IL-1beta | 2 hours |  |
| TNF-alpha | 5 minutes | Diez-Ruiz A, Tilz GP, Zangerle R et al. Soluble receptors for tumour necrosis factor in clinical laboratory diagnosis. Eur J Haematol. 1995;54(1):1-8. |
| INF-gamma | 30 minutes - 4.5 hours | Miyakawa N, Nishikawa M, Takahashi Y et al. Prolonged circulation half-life of interferon γ activity by gene delivery of interferon γ-serum albumin fusion protein in mice. J Pharm Sci. 2011;100(6):2350-7. |
| VEGF | 30 minutes | Simón-Yarza T, Formiga FR, Tamayo E et al. Vascular endothelial growth factor-delivery systems for cardiac repair: an overview. Theranostics. 2012;2(6):541-52. |
| MCP-1 | 3 hours | Cheng J, Montserrat M, Encarnacion D et al. Am J Physiol Cell Physiol 289: C959 –C970, 2005. |
| EGF | 8 minutes | Calnan DP, Fagbemi A, Berlanga-Acosta J, et al. Potency and stability of C terminal truncated human epidermal growth factor. Gut. 2000 Nov;47(5):622-7. |

**Abbreviations:** IL, interleukin; VEGF, vascular endothelial growth factor; IFN, interferon; TNF, tumor necrosis factor; MCP, monocyte chemoattractant protein; EGF, epidermal growth factor
